# Cavernous Sinus Involvement and Near Miss Mediastinitis following Mandibular Tooth Infection Treated during the COVID-19 Pandemic: Clinical Diagnosis and Treatment

**DOI:** 10.1155/2022/8650099

**Published:** 2022-07-12

**Authors:** Alessio Danilo Inchingolo, Sabino Ceci, Luisa Limongelli, Alberto Corriero, Luigi Curatoli, Daniela Azzollini, Pietro Paolo Mezzapesa, Grazia Marinelli, Giuseppina Malcangi, Giovanni Coloccia, Mario Ribezzi, Maria Massaro, Ioana Roxana Bordea, Antonio Scarano, Felice Lorusso, Nicola Brienza, Gianfranco Favia, Nicola Quaranta, Francesco Inchingolo

**Affiliations:** ^1^Department of Interdisciplinary Medicine, Section of Dental Medicine, University of Bari “Aldo Moro”, 70124 Bari, Italy; ^2^Unit of Anesthesia and Resuscitation, Department of Emergencies and Organ Transplantations, Aldo Moro University, Bari, Italy; ^3^Department Neurosciences & Sensory Organs & Musculoskeletal System, University of Bari “Aldo Moro”, 70124 Bari, Italy; ^4^Azienda Ospedaliero-Universitaria Consorziale Policlinico di Bari, Bari, Italy; ^5^Department of Oral Rehabilitation, Faculty of Dentistry, Iuliu Hațieganu University of Medicine and Pharmacy, 400012 Cluj-Napoca, Romania; ^6^Department of Innovative Technologies in Medicine and Dentistry, University of Chieti-Pescara, 66100 Chieti, Italy

## Abstract

Odontogenic infections represent a frequent condition that in some cases, if not treated promptly, can spread quickly to the rest of the body and turn into life-threatening infections. In this work, the case is reported of a 59-year-old woman, diabetic and overweight, who presented to the Odontostomatology and Otolaryngology Section of the Policlinic of Bari with mandibular tooth infection that had developed into a deep neck space infection leading to the involvement of cavernous sinuses and near mediastinum. The diagnosis, the surgical drainage of the phlegmon and removal of infection foci, appropriate control of the airways, and a correct antibiotic therapy made it possible to avoid a potentially fatal condition. Prompt management and early diagnosis of deep space neck infections, such as phlegmon and/or necrotizing fasciitis, with the auxilium of CT scans and tools such as LRINEC (Laboratory Risk Indicator for Necrotizing Fasciitis), NLR (Neutrophil-to-Lymphocyte Ratio), and LRINECxNLR scores (Laboratory Risk Indicator for Necrotizing Fasciitis and Neutrophil to Lymphocyte Ratio), are advised to evade delays and complications that could potentially worsen the patient's outcome.

## 1. Introduction

Severe deep neck abscess secondary to an odontogenic cause is often reported in the literature and is a cause for concern for the patient's life [1, 2]. Deep neck space infections (DNSIs) are bacterial infections that involve different spaces in the neck, starting from the upper aerodigestive tract [3]. Dental infections are the most common cause of DNSIs [4], followed by pharyngeal and tonsillar origin [5] and upper airway infections [6]. Generally, the source of infection is in the odontogenic periapical of the mandibular second or third molar teeth [7], which is able to invade the cortical bone. The progression of infection is often unpredictable, largely depending on the causal tooth and the head and neck anatomy of the individual. In fact, the infection may spread downward, establishing DNSI and causing mediastinitis or pericarditis, pleural empyema, jugular vein thrombosis, and septic shock. Alternatively, the infection may spread upward to the brain and cause brain abscess, cavernous sinus thrombosis, or meningitis [8, 9]. Different authors suggest the denomination descending necrotizing mediastinitis (DNM) to indicate a serious complication of the spread of cervical necrotizing fasciitis (CNF) down to the mediastinum and thorax. CNF is an aggressive and potentially lethal deep neck infection where necrosis of soft tissues such as fascia, muscle, and cervical fat is observed [10]. Nowadays, DNIs are usually more significantly decreased than in the preantibiotic era. At that time, the major part of DNIs (70%) arose from pharyngitis or tonsillitis, while today, although tonsillitis remains the most common cause in children [11], poor dental hygiene has become the most common nonsurgical cause of DNIs in adults, followed by infections of unknown origin and foreign body ingestion [12]. In this article, we report the rare case of a 59-year-old diabetic, overweight female with a bilateral phlegmonous neck collection starting from a lower right molar abscess leading to bilateral parotid gland and bilateral cavernous sinus involvement and a near miss mediastinitis. A peculiarity is the presence of gas bubbles in both cavernous sinuses and in the right half of the face.

## 2. Case Report

**Figure 1:**
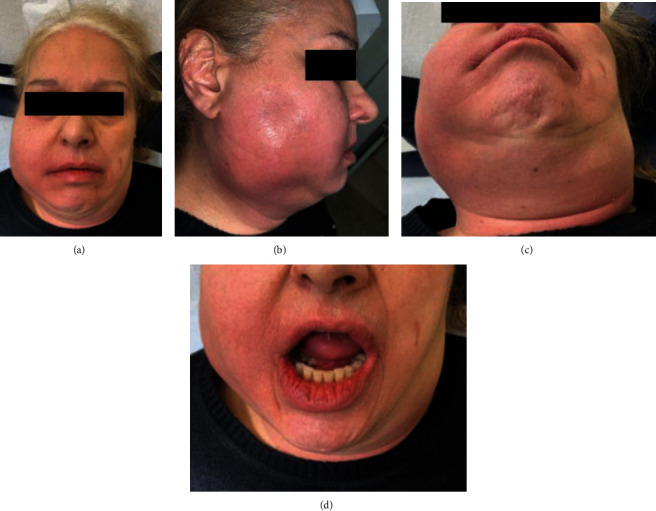
(a–d) Patient photos at ER presentation 13/02/2021: front (a); right side (b); inferior (c); open mouth (d). Evident swelling of the medial and inferior third of the right side of the face (a, b). Trismus, oedema of the tongue and mouth floor (d).

**Figure 2:**
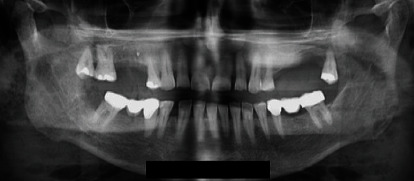
Initial orthopantomography (OPG) X-ray 13/02/2021: partial edentulism; outcomes of conservative and orthodontic therapy, prosthetic rehabilitation.

**Figure 3:**
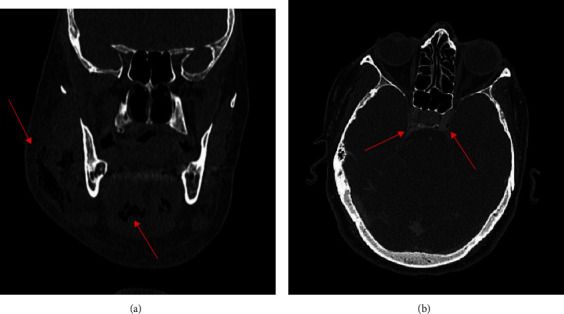
(a, b) Initial computed tomography (CT) of the head: frontal plane (a); transverse plane (b). Presence of gas bubbles in both cavernous sinuses, the right half of the face and buccal floor (red arrows) (13/02/2021).

**Figure 4:**
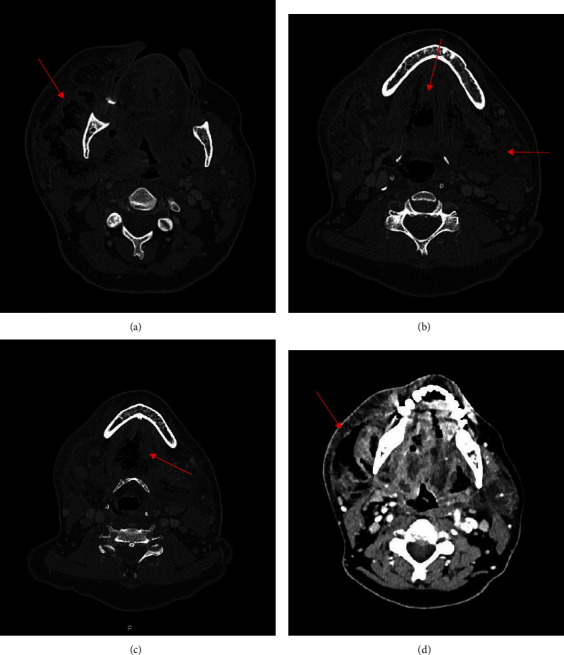
(a–d) Initial CT of the neck (transverse plane) highlighting the presence of gas bubbles (red arrows): at the right parotid gland posteriorly (a); oral floor extending up to the contralateral parotid capsule (b); sternocleidomastoid muscle medially and the thyroid space inferiorly (c); at the right parotid gland in a different cutting plane height (d) (13/02/2021).

**Figure 5:**
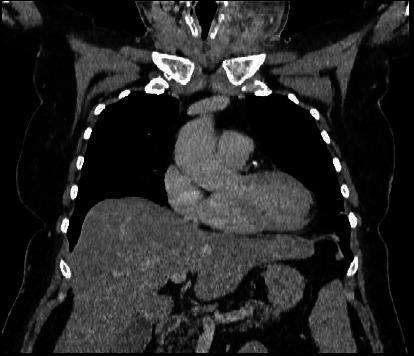
Initial CT of the chest.

**Figure 6:**
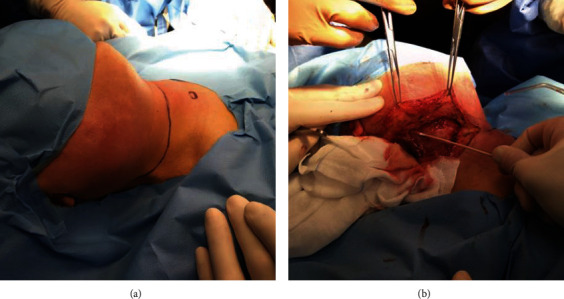
(a, b) Emergency surgery at ER: surgical procedure preparation (a); laterocervical abscess drainage (b).

The present study reports the case of a retired 59-year-old female, overweight (BMI 29.3 kg/m^2^), with decompensated type two diabetes mellitus (DM) and hypercholesterolaemia, under drug therapy (ezetimibe with simvastatin 1 tbt/day and metformin hydrochloride 1000 mg 1 tbt twice a day), no coronary artery disease or herpetic virus liver disease, previous appendectomy surgery under general anaesthesia, and common childhood rashes. The patient was treated during the COVID-19 period, and all indicated measures according to the scientific literature were taken to protect all medical workers and the patient. Seven days prior to emergency room (ER) admission, she reported swelling in the right mandibular region which showed progressive volumetric increase with spontaneous and on-palpation pain. She started a nonbetter-specified antibiotic therapy with no benefits at all so that a subsequent dysphonia and liquid and solid dysphagia developed. On ER physical examination, the patient presented a voluminous right half-face abscess, inability to speak correctly due to trismus, and oedema of the tongue and mouth floor (Figure 1). Optical fiber video exploration of the upper respiratory tract showed mucosal oedema and hyperaemia of both the hypopharynx and the larynx. Complete blood count and laboratory tests (the most relevant values are reported in Table 1), Sars-CoV2 swab (negative), orthopantomography (OPG) X-ray (Figure 2), computed tomographies (CT) of the head-neck (Figures 3 and 4), and chest (Figure 5) were performed. Skull CT underlined the presence of gas bubbles in both the cavernous sinuses, possible odontogenic focus on tooth 4.7, part of a prosthetic bridge (4.7–4.5); neck CT was positive for huge phlegmon and gas bubbles affecting the right half of the face and platysma, the right parotid gland posteriorly, extending up to the contralateral parotid capsule and sternocleidomastoid muscle medially and the thyroid space inferiorly; chest CT showed no pathological findings. Emergency surgical laterocervical abscess drainage appeared to be mandatory and was performed together with the homolateral submandibular gland sialoadenectomy. The patient also underwent a surgical tracheostomy, according to the previous optic fibre video evaluation, in order to secure the airways and ensure proper ventilation (Figure 6). Antibiotic therapy with Teicoplanin 400 mg and Levofloxacin 500 mg was prescribed (Targosid 400 (Teicoplanina) mg Endovein at 12 : 00 and 22 : 00 with Rocefin (Ceftriaxone) 2 g Endovein at 8 : 00 and 20 : 00, and Desometasone 8 MG at 8 : 00 and 22 : 00 + gastroprotectives.)

**Figure 7:**
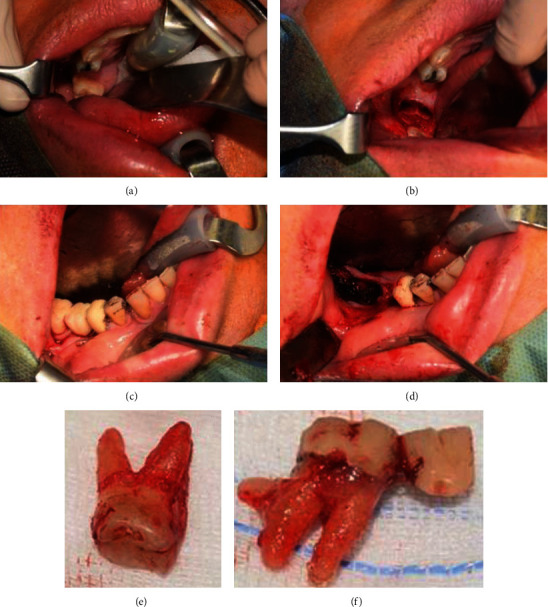
(a–f) Extraction of 1.7 (a, b); extraction of 4.7 plus pontic (c, d); extracted 1.7 (e) and 4.7 (f).

The day after the emergency surgery, neurosurgical signs (negative) and infectious disease (new antibiotic therapy: piperacillin/tazobactam 4.5 g and linezolid 600 mg) consultations were requested. Urgent head and neck-chest computed tomography (CT) scans were performed. Presence of bilateral gas bubbles in correspondence to the cavernous sinuses was revealed. At the level of the neck, a large phlegmonous collection was present with a considerable contextual air share located mainly in the right half of the face, the platysma, and the ipsilateral parotid gland capsule. Medially, it extended to the buccal floor to the left submandibular lodge and the sternocleidomastoid fascia. C-reactive protein value was reduced but still high. On the third day of hospitalization, the patient was admitted to the intensive care unit because septic shock had developed due to the patient requiring vasopressors to maintain a mean arterial pressure higher than 65 mmHg and presenting a serum lactate level greater than 2 mmol/L. SOFA score was 9, and fever was present (38.2 degrees C). Neck-chest CT showed increased phlegmonous collection, especially on the left side (extended to the left parotid gland), on the anterior region of the neck, and down to the superior mediastinal region in the retrosternal site. Head CT revealed almost complete reabsorption of the gaseous collections at the level of the cavernous sinuses on both sides; only a small gas bubble remained in the right cavernous sinus. Emphysema in the neck was also moderately reabsorbed. There were no signs of venous thrombosis of the cerebral vessels or neck. Thoracic surgery consultation revealed no need for emergency surgery and neck-thorax CT with contrast medium after 48 hours and revaluation. The patient proceeded to surgery (Figure 7). After local anaesthesia (mepivacaine 3%) without vasoconstrictor, tooth 1.7 was removed because of a periapical lesion with necrosis; afterwards, the prosthetic bridge 4.5-4.7 was sectioned with rotating instruments in order to save the 4.5. Tooth 4.7 was extracted together with the pontic 4.6. Since a radiopacity spot in area 4.8 was seen on the skull-CT, a surgical revision in this site was also carried out. Alveolar curettage was performed using Volkmann spoons, and two specimens of exudate, collected in the postextraction site (4.7) and in 4.8 area, were sent for histological and microbiological examination; after washing with physiological solution and rifamicine topic antibiotic, haemostis with oxidized regenerated cellulose gauzes (Tabotamp®) was performed.

**Figure 8:**
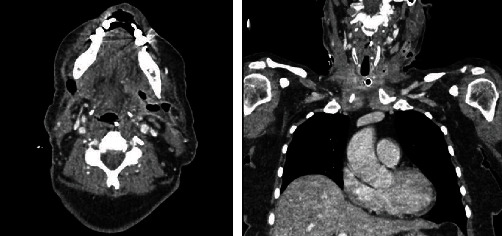
Final CT neck and chest 01/03/2021.

The day after surgery, the patient was kept sedated and under controlled mechanical ventilation; SOFA score dropped from 9 to 8, and haemodynamic stability was achieved through vasopressors (noradrenaline). Fever was still present. In the following days, as the SOFA score dropped to 0 and discontinuation from amine support transpired, fever resolved, and the patient initiated successful weaning that resulted in disconnection from the ventilator on the 9th day of hospitalization. ENT (ear, nose, and throat) consultation revealed a thorough surgical treatment and suggested surgical wound dressing, washing with H_2_O_2_ and physiological solution through the drains. In addition, dentistry and thoracic surgery consultations were required. A new neck-thorax CT with contrast medium showed unchanged bilateral neck abscess and involvement of the anterior part of the neck down to the superior mediastinal region in the retrosternal site that did not require surgical treatment. After 10 days of hospitalization, chest X-ray did not show any variation compared to the previous evaluation, and the patient's condition was stable, being neurologically well oriented, hemodynamically stable, and with good gas exchange. On the 12th day, the patient was moved to the ENT ward. Patient discharge took place approximately 1 month after performing neck and chest CTs (Table 2 and Figure 8).

**Figure 9:**
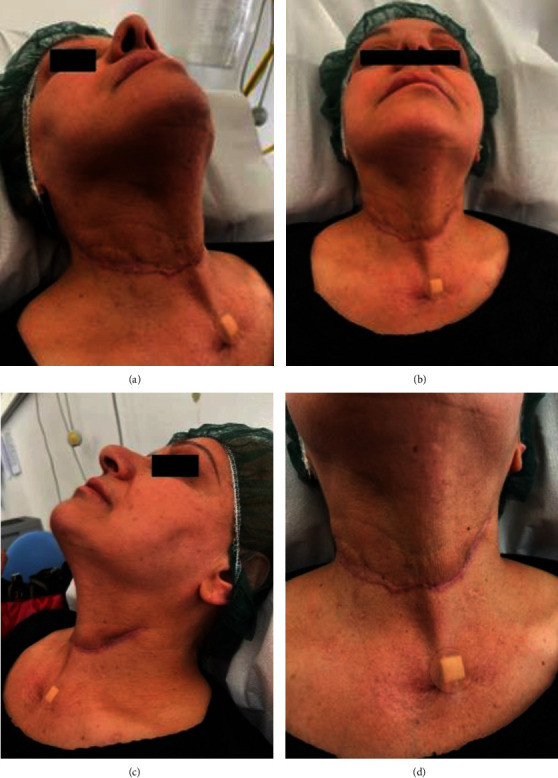
(a–d) Final patient photos: right side (a); front (b); left side (c); neck scar detail (d). Presence of scarring outcomes on the neck; absence of swelling of the face and neck.

**Figure 10:**
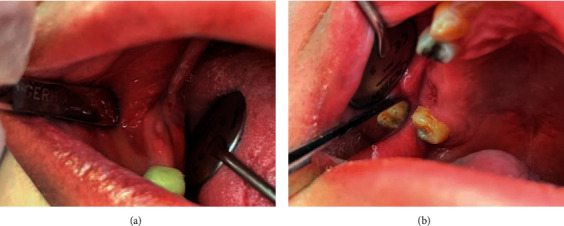
(a, b) Final intraoral photos: after extraction site of 4.7 (a); after extraction site of 1.7 (b). Good tissue healing on the extraction sites of 4.7 (blue arrow) and 1.7 (green arrow).

**Figure 11:**
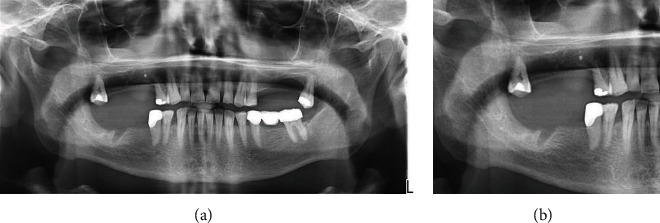
(a, b) Final OPG X-ray (17/06/2021): no relevant findings on the extraction sites (magnification (b)).

Check-up 3 months after discharge revealed no externally visible swelling (Figure 9) and a good intraoral tissues healing (Figure 10). Control OPG (Figure 11); CTs of head-neck and chest were requested and checked.

Neck-chest CT (13/02/2021): presence of bilateral gas bubbles in correspondence to the cavernous sinuses. At the level of the neck, a large phlegmonous collection with a considerable contextual air share located mainly in the right half face, the platysma, and the ipsilateral parotid gland capsule. Medially, it extended to the buccal floor to the left submandibular lodge and the sternocleidomastoid fascia.

Chest X-ray (bedridden) (14/02/2021): presence of tracheostomy tube with distal end in place; raised right hemidiaphragm with ipsilateral basal dysventilation; heart substantially within the limits.

CT neck-chest (15/02/2021): increase of the known phlegmon, especially on the left side (where it also extends to the left parotid gland) and also to the anterior region of the neck and down to the superior mediastinal region in the retrosternal site.

Cranial CT (15/02/2021): almost complete reabsorption of the gaseous collections at the level of the cavernous sinuses on both sides; currently, only a small gas box remains in the right cavernous sinus. Emphysema in the neck is also moderately reabsorbed; no signs of venous thrombosis of the cerebral vessels and neck.

Ct neck and thorax with contrast medium (17/02/2021): the known abscess collection in the neck bilaterally unchanged, especially on the left. It also extends to the anterior region of the neck and down to the superior mediastinal region in the retrosternal site; filiform the lumen of the pharynx and larynx. The pharyngolaryngeal spaces are obliterated. The remaining finds unchanged.

Chest X-ray bedridden (22/02/2021): no variation compared to the previous.

The written informed consent for the study and case publishing was obtained from the patient recruited.

## 3. Results and Discussion

Infections of dental origin are common and are well-controlled by antibiotic prescription. Nevertheless, when not controlled, these infections could spread into the cervical and maxillofacial region, thus risking a myriad of possible complications including Ludwig's angina, respiratory obstruction, facial deep neck abscesses, cellulitis, NCF, aspiration pneumonia, septicaemia, thoracic empyema, descending mediastinitis, pleuropulmonary suppuration, brain abscess, endocarditis, pericarditis, pneumothorax, jugular thrombophlebitis and abscess of the carotid sheath, hematogenous dissemination to distant organs, fulminant state of disseminated intravascular coagulation (DIC), and coagulation abnormalities which could all eventually result in mortality [9, 13, 14]. DNSIs are infections involving the pharyngeal and neck spaces [15–19]. The most frequent sources of infection are oral cavity, in particular teeth, and pharynx [6, 20], although in 25% of cases, there is no clear source [5]. The location frequency is as follows: 36% submandibular, 13% sublingual, 12% parapharyngeal, and 3% retropharyngeal [5]. Bacteria from different locations such as oral cavity, pharynx, blood, and tonsils can cause abscesses and phlegmon (a suppurative inflammation of the subcutaneous connective tissue) [21], spreading along the connective tissues and fascial spaces. DNSIs are a quite common and potentially life-threating entity, related to high rates of hospitalisation, complications, and eventually death. Prompt medical, and often surgical, treatment is mandatory in order to avoid fatal outcomes [22]. If a correct diagnosis and surgical treatment are carried out within the first twelve hours, the mortality rate drops [23, 24]. A first strength in the treatment of the patient in this case report was certainly the rapid laterocervical surgical drainage, tracheostomy, and administration of a correct and adequate antibiotic therapy. In fact, treatment of these infections is complex and includes broad-spectrum antibiotics, airway management (including tracheostomy), and surgical intervention. Furthermore, a surgical drainage of the abscess may be necessary in the first place [4, 6]. Life-threatening complications are airway compromise, mediastinitis, pericarditis, intracranial involvement, and arterial erosion [25–29]. Another grave complication is descending necrotizing mediastinitis (DNM) [1], a serious and progressive infection involving the neck and the chest, in which an odontogenic, pharyngeal, or cervical infection spreads rapidly through the subcutaneous tissue and cervical fascia to the thoracic cavity causing tissue necrosis, with a high death rate (10-40%) by sepsis and organic failure if not treated quickly and appropriately [30, 31]. Almost 60-70% of DNM cases are due to descending odontogenic infections, first of all, when they are caused by second and third lower molar necrosis [32]. Therefore, a second strength in the present case is certainly the avoidance of fatal mediastinitis, a “near miss” mediastinitis, because the patient was treated quickly and properly. Another important aspect to consider is the presence of comorbidities or other clinical conditions. The patient reported in this article suffers from diabetes and is overweight. DM, liver cirrhosis, alcoholism, hypertension, chronic renal insufficiency, and malignancy are necrotizing fasciitis (NF) predisposing factors [33]. DM is the most frequently encountered comorbidity, and it greatly increases the risk of complications of DNSI and mortality [34]. In a study of 263 cases, a high number of subjects with DNSI had associated systemic diseases, such as primary arterial hypertension, coronary heart disease, DM, and HIV infection [4, 35]. The impact of body mass index (BMI) in the development of DNSI is remarkable: in fact, if it increases, it is clear that acute phlegmonous laryngitis as etiological agent, phlegmon of the neck as localization, and complications augment [35].

A notable aspect in this case was the presence of gas bubbles at the level of the cavernous sinus, as highlighted by CT. This scan is a very accurate tool, with 79% sensitivity and 94% specificity, which is why it is requested on patient admission and during hospitalization [36, 37]. Presence of gas bubbles resulting from bacterial fermentation is not uncommon in a wide range of important infections. The microbiota is also an important aspect that can influence the evolution of a patient. A particular pathological event, necrotizing fasciitis (NF), a term introduced by Wilson in 1952 [38] to indicate an inflammatory process of soft tissues, determining a fascial necrosis, can allow an easy and wide spread of the infection along the laterocervical bands and thus through locus minoris resistentiae, reaching the mediastinum and causing descending necrotizing mediastinitis (DNM). The greatest incidence of NF is in the 30-50 age group, although no age group is excluded [39–46], and it is characterized by a high mortality rate [3]. The oral cavity is the most frequent starting location of the infection [39, 41, 47–55]. Cervical necrotizing fasciitis (CNF) indicates necrosis involvement of the neck tissues, especially less vascularized tissues, i.e., fascia, but also fat and muscles [46, 56, 57]. Only 3.5% of DNSIs are CNF, but the mortality rate is 7-22%, 41% if there is thoracic involvement, and 64% with DNM and complications caused by sepsis [58]. There are 2 different forms of CNF: suppurative and gaseous. The first one is characterized by purulent fluid collection; the second by gas formation [10]. Sakai et al. [59] analysed six patients with DNM caused by pharyngolaryngeal and odontogenic infection which required broad-spectrum antibiotic therapy, aggressive drainage, and thoracic surgery. Four of these patients had polymicrobial infections (aerobic and anaerobic bacteria including *Streptococcus anginosus* group SAG) and all of them showed gas bubbles on chest TC. Ng et al. [60] reported the case of a 66-year-old man with Emphysematous aortitis from Clostridial infection of the thoracic aorta with presence of gas bubbles along the aortic wall. These findings were visible in the CT scan of the chest. After proliferation in necrotic or anoxic areas, Clostridia can release toxins and enzymes (hyaluronidase and hemolysin), producing gas gangrene infection. In another article, Xing et al. [61] described a case of emphysematous pyelonephritis (EPN), a lethal necrotic infection of the kidney with collection of gas in the renal parenchyma, and/or perinephric tissues [62]. Point-of-care ultrasound (POCUS) detected gas bubbles in the hepatorenal space. *E. coli* and other *Enterobacteriaceae* are the most frequent causes of EPN, and they produce gas by fermentation of glucose and lactate in necrotic tissues [63]. Richards et al. [64] reported a 64-year-old woman who presented to the emergency department with a 6-day history of dental pain and significant right-sided facial swelling. A panoramic radiograph showed periapical radiolucencies associated with her carious lower right first premolar (LR5) and second molar (LR7) which led to a diagnosis of rapidly progressing odontogenic abscess. CT revealed an extensive abscess with gas pockets adjacent to the right mandible which was indicative of the severity of the infection. In the case of the patient mentioned in the present article, the microbiological examination of the exudate taken from the postextraction site was performed, which gave a negative result. In literature, the evaluation of exudate from the abscess site or from the gangrenous necrosis region frequently provides negative feedback [65, 66] and is not considered of fundamental importance. On other occasions, it provides uncertain results, multiple etiology [42, 47, 50, 51, 65–72], or the finding of saprophytic or symbiont microorganisms. It can be useful to administer a specific antibiotic treatment if it is possible to highlight certain bacterial strains and/or fungi. An additional strength in the management of this clinical case was the empirical antibiotic therapy, developed by combining an antibiotic active against gram negative and one active against gram positive as this is frequently found in the literature [49, 66, 67, 73–77]. It is important to define and use some reliable tools to quickly detect and identify DNSI complications such as CNF, DNM, and systemic sepsis. There are some clinical signs and symptoms useful for identifying CNF but they have only 67% of sensitivity [78]. On the other hand, there are no specific laboratory studies proven to be predictive of the NF diagnosis [79], although Fiorella et al. propose the use of Laboratory Risk Indicator for Necrotizing Fasciitis (LRINEC) score (Table 3), the neutrophil-to-lymphocyte ratio (NLR), and LRINEC × NLR scores as predictive tools to early evaluate septic complications and the risk of CNF during DNSI. Indicator LRINEC×NLR was proposed by Fiorella et al. to enhance the predictive value of the analysis [80]. LRINEC score was introduced by Wong et al. to help clinicians access for NF checking on serum: white blood cell count, haemoglobin, sodium, glucose, creatinine, and C-reactive protein. NLR has an important meaning especially during the initial and last phases of sepsis [81]. It was initially proposed by Baglam et al. as a predictive factor for DNSI [82]. Patient LRINEC score, calculated on the parameters shown in Table 2, was 6. This indicated a medium risk of developing NF according to Wong et al. and Chen et al. [58, 81] with 94% of sensitivity and specificity according to Sandner et al. [36]. NLR score was 8.9 predicting systemic septic involvement (sensitivity and specificity rate of 74.2% and 61.5% above value of 8.2) and LRINEC×NLR score 53.4 indicating an important risk of CNF having a sensitivity of 90.9% above values of 43.5 according to Fiorella et al. [80]. In this case report, it is possible to see how failure to apply the LRINEC score to early detect a rapidly developing necrotizing failure could potentially lead to either a mediastinitis or a life-threatening infection in the cranial fossae: the patient should have been screened in the emergency room and correctly directed into an intensive care ward rather than ENT ward to establish proper care, which happened only after 48 hours from hospitalization.

## 4. Conclusions

Early treatment of any odontogenic infection should be taken care of since a missed or late diagnosis of deep space neck infection could potentially cause serious complications. Surgical drainage within the first hours, early extraction of the odontogenic focuses, proper airway management, and broad-spectrum antibiotic treatment decreased the number of hospitalization days and the recovery period, without increasing complications.

Take home messages:

1. Correct early treatment of odontogenic infections
2. LRINEC, NLR, and LRINEC×NLR scores should be assessed quickly to direct the patient to the best diagnostic/therapeutic path
3. Collaboration between different specialities regarding the treatment of this type of case is mandatory

## Figures and Tables

**Table 1:** Most relevant values of the complete blood count.

	Laboratory tests
Uraemia	105 mg/dL
Glycaemia	414 mg/dL
eGFR	68 mL/min
Albumin	2.3 g/dL
CRP	389 mg/L
Fibrinogen	1279 mg/dL
D-dimers	1068 *μ*g/L
pO2	70 mmHg

**Table 2:** Data regarding the patient evaluation.

Study timeline	
13-02-2021	E.R. admission, emergency surgery, airway management
15-02-2021	I.C.U. admission: oral surgery procedure
24-02-2021	E.N.T. admission
15-03-2021	Discharge
17-06 2021	Check-up

**Table 3:** LRINEC scores.

Variable, unit	Score
C-reactive protein (CRP), mg/L	
<150	0
≥150	4
Haemoglobin, g/dL	
>13.5	0
11-13.5	1
<11	2
Total white cell count, per mm^3^	
<15	0
15-25	1
>25	2
Sodium, mmol/L	
≥135	0
<135	2
Creatinine, *μ*mol/L	
≤141	0
>141	2
Glucose, mg/dL	
≤180	0
>180	1

LRINEC scores: low risk (probability less than 50%) for LRINEC-score 5; medium risk (probability of 50-75%) for LRINEC-score between 6 and 7; high risk (probability more than 75%) for LRINEC − score ≥ 8.

## Data Availability

All experimental data to support the findings of this study are available upon request by contacting the corresponding author. The authors have annotated the entire data building process and empirical techniques presented in the paper. The data underlying this article are not freely available by agreement with our partners to protect their confidentiality.

## References

[1] Tormes A. K., De Bortoli M. M., Júnior R. M., Andrade E. S. (2018). Management of a severe cervicofacial odontogenic infection. *The Journal of Contemporary Dental Practice*.

[2] Kinzer S., Pfeiffer J., Becker S., Ridder G. J. (2009). Severe deep neck space infections and mediastinitis of odontogenic origin: clinical relevance and implications for diagnosis and treatment. *Acta Oto-Laryngologica*.

[3] Priyamvada S., Motwani G. (2019). A study on deep neck space infections. *Indian Journal of Otolaryngology and Head & Neck Surgery*.

[4] Prabhu S., Nirmalkumar E. (2019). Acute fascial space infections of the neck: 1034 cases in 17 years follow up. *Annals of Maxillofacial Surgery*.

[5] Velhonoja J., Lääveri M., Soukka T., Irjala H., Kinnunen I. (2020). Deep neck space infections: an upward trend and changing characteristics. *European Archives of Oto-Rhino-Laryngology*.

[6] Huang T.-T., Liu T.-C., Chen P.-R., Tseng F.-Y., Yeh T.-H., Chen Y.-S. (2004). Deep neck infection: analysis of 185 cases. *Head & Neck*.

[7] Boscolo-Rizzo P., Stellin M., Muzzi E. (2012). Deep neck infections: a study of 365 cases highlighting recommendations for management and treatment. *European Archives of Oto-Rhino-Laryngology*.

[8] Pesis M., Bar-Droma E., Ilgiyaev A., Givol N. (2019). Deep neck infections are life threatening infections of dental origin: a presentation and management of selected cases. *The Israel Medical Association Journal*.

[9] Montemurro N., Perrini P., Marani W. (2021). Multiple brain abscesses of odontogenic origin. May oral microbiota affect their development? A review of the current literature. *Applied Sciences*.

[10] Karkas A., Chahine K., Schmerber S., Brichon P.-Y., Righini C. A. (2010). Optimal treatment of cervical necrotizing fasciitis associated with descending necrotizing mediastinitis. *British Journal of Surgery*.

[11] Blankson P.-K., Parkins G., Boamah M. O. (2019). Severe odontogenic infections: a 5-year review of a major referral hospital in Ghana. *The Pan African Medical Journal*.

[12] Marioni G., Rinaldi R., Staffieri C. (2008). Deep neck infection with dental origin: analysis of 85 consecutive cases (2000-2006). *Acta Oto-Laryngologica*.

[13] Opitz D., Camerer C., Camerer D.-M. (2015). Incidence and management of severe odontogenic infections--a retrospective analysis from 2004 to 2011. *Journal of Cranio-Maxillo-Facial Surgery*.

[14] Zawiślak E., Nowak R. (2021). Odontogenic head and neck region infections requiring hospitalization: an 18-month retrospective analysis. *BioMed Research International*.

[15] Coticchia J. M., Getnick G. S., Yun R. D., Arnold J. E. (2004). Age-, site-, and time-specific differences in pediatric deep neck abscesses. *Archives of Otolaryngology – Head & Neck Surgery*.

[16] Kirse D. J., Roberson D. W. (2001). Surgical management of retropharyngeal space infections in children. *The Laryngoscope*.

[17] McClay J. E., Murray A. D., Booth T. (2003). Intravenous antibiotic therapy for deep neck abscesses defined by computed tomography. *Archives of Otolaryngology – Head & Neck Surgery*.

[18] Miller W. D., Furst I. M., Sàndor G. K. B., Keller M. A. (1999). A prospective, blinded comparison of clinical examination and computed tomography in deep neck infections. *The Laryngoscope*.

[19] Nagy M., Pizzuto M., Backstrom J., Brodsky L. (1997). Deep neck infections in children: a new approach to diagnosis and treatment. *The Laryngoscope*.

[20] Koç A. K., Alakhras W. M., Acıpayam H., Koçak H. E., Kayhan F. T. (2016). Seven years of experience in 160 patients with deep neck infection. *Imaging (MRI)*.

[21] Simion L., Dumitru S. (2018). Phlegmon of the oral floor. Contradictions in diagnosis and treatment. *The Moldovan Medical Journal*.

[22] Vieira F., Allen S. M., Stocks R. M. S., Thompson J. W. (2008). Deep neck infection. *Otolaryngologic Clinics of North America*.

[23] Muhammad J. K., Almadani H., Al Hashemi B. A., Liaqat M. (2015). The value of early intervention and a multidisciplinary approach in the management of necrotizing fasciitis of the neck and anterior mediastinum of odontogenic origin. *Journal of Oral and Maxillofacial Surgery*.

[24] Cruz Toro P., Callejo Castillo À., Tornero Saltó J., González Compta X., Farré A., Maños M. (2014). Cervical necrotizing fasciitis: report of 6 cases and review of literature. *European Annals of Otorhinolaryngology, Head and Neck Diseases*.

[25] Chan W.-L., Fernandes V. B., Carolan M. G. (1999). Retropharyngeal abscess on a Ga-67 scan: a case report. *Clinical Nuclear Medicine*.

[26] Abba Y., Hassim H., Hamzah H., Noordin M. M. (2015). Antiviral activity of resveratrol against human and animal viruses. *Advances in Virology*.

[27] Kim H.-J., Park E. D., Kim J. H., Hwang E. G., Chung S. H. (1997). Odontogenic versus nonodontogenic deep neck space infections: CT manifestations. *Journal of Computer Assisted Tomography*.

[28] Ursic C. M., Shah S. V., Kaviani A. (2001). Neck abscess after blunt cervical trauma. *The Journal of Trauma: Injury, Infection and Critical Care*.

[29] Waggie Z., Hatherill M., Millar A., France H., Van Der Merwe A., Argent A. (2002). Retropharyngeal abscess complicated by carotid artery rupture. *Pediatric Critical Care Medicine*.

[30] Prado-Calleros H. M., Jiménez-Fuentes E., Jiménez-Escobar I. (2016). Descending necrotizing mediastinitis: systematic review on its treatment in the last 6 years, 75 years after its description. *Head & Neck*.

[31] Benedetto C., Tanzariello V. N., Militi A. (2020). Catastrophic descending necrotizing mediastinitis of the anterior and posterior compartments: a case report. *Radiology Case Reports*.

[32] Sakamoto H., Aoki T., Kise Y., Watanabe D., Sasaki J. (2000). Descending necrotizing mediastinitis due to odontogenic infections. *Oral Surgery, Oral Medicine, Oral Pathology, Oral Radiology, and Endodontology*.

[33] Wang J.-M., Lim H.-K. (2014). Necrotizing fasciitis: eight-year experience and literature review. *Brazilian Journal of Infectious Diseases*.

[34] Bross-Soriano D., Arrieta-Gómez J. R., Prado-Calleros H., Schimelmitz-Idi J., Jorba-Basave S. (2004). Management of Ludwig’s angina with small neck incisions: 18 years experience. *Otolaryngology and Head and Neck Surgery*.

[35] Adoviča A., Veidere L., Ronis M., Sumeraga G. (2017). Deep neck infections: review of 263 cases. *Otolaryngologia Polska*.

[36] Sandner A., Moritz S., Unverzagt S., Plontke S. K., Metz D. (2015). Cervical necrotizing fasciitis--the value of the laboratory risk indicator for necrotizing fasciitis score as an Indicative parameter. *Journal of Oral and Maxillofacial Surgery*.

[37] Thomas A. J., Meyer T. K. (2012). Retrospective evaluation of laboratory-based diagnostic tools for cervical necrotizing fasciitis. *The Laryngoscope*.

[38] Wilson B. (1952). Necrotizing fascitis. *The American Surgeon*.

[39] Valko P. C., Barret S. M., Campbell J. P. (1990). Odontogenic cervical necrotizing fasciitis. *Annals of Emergency Medicine*.

[40] Gillis A. R., Gillis T. M. (1992). Necrotizing cervical fasciitis of unknown origin. *The Journal of Otolaryngology*.

[41] Kantu S., Har-El G. (1997). Cervical necrotizing fasciitis. *The Annals of Otology, Rhinology, and Laryngology*.

[42] Isaacs L. M., Kotton B., Peralta M. M. (1993). Fatal mediastinal abscess from upper respiratory infection. *Ear, Nose, & Throat Journal*.

[43] Chua H. K., Segar C. B., Krishnan R., Ho C. K. (2002). Cervical necrotising fasciitis consequent to mastoid infection. *The Medical Journal of Malaysia*.

[44] Safak M. A., Haberal I., Kiliç D., Göçmen H. (2001). Necrotizing fasciitis secondary to peritonsillar abscess: a new case and review of eight earlier cases. *Ear, Nose, & Throat Journal*.

[45] Ndukwe K. C., Fatusi O. A., Ugboko V. I. (2002). Craniocervical necrotizing fasciitis in Ile-Ife, Nigeria. *British Journal of Oral and Maxillofacial Surgery*.

[46] Skitarelić N., Mladina R., Morović M. (2003). Cervical necrotizing fasciitis: sources and outcomes. *Infection*.

[47] Greinwald L. J. H., Wilson J. F., Haggerty P. G. (1995). Peritonsillar abscess: an unlikely cause of necrotizing fasciitis. *The Annals of Otology, Rhinology, and Laryngology*.

[48] Balcerak R. J., Sisto J. M., Bosack R. C. (1988). Cervicofacial necrotizing fasciitis: report of three cases and literature review. *Journal of Oral and Maxillofacial Surgery*.

[49] Helmy A. S., Salah M. A., Nawara H. A., Khatab H., Khalaf H. A., Abd el-Maguid N. (1997). Life-threatening cervical necrotizing fasciitis. *Journal of the Royal College of Surgeons of Edinburgh*.

[50] Maisel R. H., Karlen R. (1994). Cervical necrotizing fasciitis. *The Laryngoscope*.

[51] De Backer T., Bossuyt M., Schoenaers J. (1996). Management of necrotizing fasciitis in the neck. *Journal of Cranio-Maxillofacial Surgery*.

[52] Dohan Ehrenfest D. M., Del Corso M., Inchingolo F., Charrier J.-B. (2010). Selecting a relevant in vitro cell model for testing and comparing the effects of a Choukroun's platelet-rich fibrin (PRF) membrane and a platelet-rich plasma (PRP) gel: tricks and traps. *Oral Surgery, Oral Medicine, Oral Pathology, Oral Radiology, and Endodontics*.

[53] Inchingolo F., Martelli F. S., Gargiulo Isacco C. (2020). Chronic periodontitis and immunity, towards the implementation of a personalized medicine: a translational research on gene single nucleotide polymorphisms (SNPs) linked to chronic oral dysbiosis in 96 Caucasian patients. *Biomedicines*.

[54] Charitos I. A., Ballini A., Bottalico L. (2020). Special features of SARS-CoV-2 in daily practice. *World Journal of Clinical Cases*.

[55] Pham V. H., Gargiulo Isacco C., Nguyen K. C. D. (2020). Rapid and sensitive diagnostic procedure for multiple detection of pandemic Coronaviridae family members SARS-CoV-2, SARS-CoV, MERS-CoV and HCoV: a translational research and cooperation between the Phan Chau Trinh University in Vietnam and University of Bari ‘Aldo Moro’ in Italy. *European Review for Medical and Pharmacological Sciences*.

[56] Lin C., Yeh F.-L., Lin J.-T. (2001). Necrotizing fasciitis of the head and neck: an analysis of 47 cases. *Plastic and Reconstructive Surgery*.

[57] Mohammedi I., Ceruse P., Duperret S., Vedrinne J.-M., Boulétreau P. (1999). Cervical necrotizing fasciitis: 10 years’ experience at a single institution. *Intensive Care Medicine*.

[58] Chen L. L., Fasolka B., Treacy C. (2020). Necrotizing fasciitis: a comprehensive review. *Nursing*.

[59] Sakai T., Sano A., Azuma Y., Koezuka S., Otsuka H., Iyoda A. (2021). Streptococcus anginosus group infection as a predictor for the progression of descending necrotizing mediastinitis. *Annals of Palliative Medicine*.

[60] Ng T. G., Trivedi U., Shah K., Maldjian P. (2021). Clostridial mycotic aneurysm leading to emphysematous aortitis. *Cureus*.

[61] Xing Z.-X., Yang H., Zhang W. (2021). Point-of-care ultrasound for the early diagnosis of emphysematous pyelonephritis: a case report and literature review. *WJCC*.

[62] Song Y., Shen X. (2020). Diabetic ketoacidosis complicated by emphysematous pyelonephritis: a case report and literature review. *BMC Urology*.

[63] Ubee S. S., McGlynn L., Fordham M. (2011). Emphysematous pyelonephritis. *BJU International*.

[64] Richards C., Walker T. W., Girgis S., Colbert S. (2020). ‘Bubble sign’: gas-forming bacteria from an odontogenic infection. *BML Case Reports*.

[65] Mathieu D., Neviere R., Teillon C., Chagnon J. L., Lebleu N., Wattel F. (1995). Cervical necrotizing fasciitis: clinical manifestations and management. *Clinical Infectious Diseases*.

[66] Gidley P., Ghorayeb B., Stiernberg C. (1997). Contemporary management of deep neck space infections☆☆☆. *Otolaryngology-Head and Neck Surgery*.

[67] Rapoport Y., Himelfarb M. Z., Zikk D., Bloom J. (1991). Cervical necrotizing fasciitis of odontogenic origin. *Oral Surgery, Oral Medicine, Oral Pathology*.

[68] Freischlag J. A., Ajalat G., Busuttill R. W. (1985). Treatment of necrotizing soft tissue infections: the need for a new approach. *The American Journal of Surgery*.

[69] Krespi Y. P., Lawson W., Blaugrund S. M., Biller H. F. (1981). Massive necrotizing infections of the neck. *Head & Neck*.

[70] Moss R. M., Kunpittaya S., Sorasuchart A. (1990). Cervical necrotizing fasciitis: an uncommon sequela to dental infection. *Annals of Otology, Rhinology & Laryngology*.

[71] Santacroce L., Charitos I. A., Ballini A. (2020). The human respiratory system and its microbiome at a glimpse. *Biology*.

[72] Ballini A., Gnoni A., De Vito D. (2019). Effect of probiotics on the occurrence of nutrition absorption capacities in healthy children: a randomized double-blinded placebo-controlled pilot study. *European Review for Medical and Pharmacological Sciences*.

[73] Bahna M., Canalis R. F. (1980). Necrotizing fasciitis (streptococcal gangrene) of the face: report of a case and review of the literature. *Archives of Otolaryngology-Head and Neck Surgery*.

[74] Beerens A. J. F., Bauwens L. J., Leemans C. R. (1999). A fatal case of craniofacial necrotizing fasciitis. *European Archives of Oto-Rhino-Laryngology*.

[75] Lang M. E., Vaudry W., Robinson J. L. (2003). Case report and literature review of late-onset group B streptococcal disease manifesting as necrotizing fasciitis in preterm infants: is this a new syndrome?. *Clinical Infectious Diseases*.

[76] Feinerman I. L., Tan H. K. K., Roberson D. W., Malley R., Kenna M. A. (1999). Necrotizing fasciitis of the pharynx following adenotonsillectomy. *International Journal of Pediatric Otorhinolaryngology*.

[77] Skorina J., Kaufman D. (1995). Necrotizing fasciitis originating from pinna perichondritis. *Otolaryngology-Head and Neck Surgery*.

[78] Goldstein E. J. C., Anaya D. A., Dellinger E. P. (2007). Necrotizing soft-tissue infection: diagnosis and management. *Clinical Infectious Diseases*.

[79] Moskowitz E., Schroeppel T. (2018). Necrotizing fasciitis following abdominal gunshot wound. *Trauma Surgery & Acute Care Open*.

[80] Fiorella M. L., Greco P., Madami L. M., Giannico O. V., Pontillo V., Quaranta N. (2020). New laboratory predictive tools in deep neck space infections. *Acta Otorhinolaryngologica Italica*.

[81] Wong C.-H., Khin L.-W., Heng K.-S., Tan K.-C., Low C.-O. (2004). The LRINEC (laboratory risk indicator for necrotizing fasciitis) score: a tool for distinguishing necrotizing fasciitis from other soft tissue infections. *Critical Care Medicine*.

[82] Baglam T., Binnetoglu A., Yumusakhuylu A. C., Gerin F., Demir B., Sari M. (2015). Predictive value of the neutrophil-to-lymphocyte ratio in patients with deep neck space infection secondary to acute bacterial tonsillitis. *International Journal of Pediatric Otorhinolaryngology*.

